# Hantavirus Outbreak Caused by Andes Virus Linked to International Cruise Ship Travel — United States, 2026

**DOI:** 10.15585/mmwr.mm7543a1

**Published:** 2026-11-05

**Authors:** Ozair H. Naqvi, Mary J. Choi, Katrin S. Sadigh, Rita Austin, Bryon P. Backenson, Harveen Bergquist, Alexis Beyer, David Blythe, Sarah Bowden, Andrew T. Boyd, Chris K. Brown, Bryan F. Buss, Thomas G. Calogrides, Wesley R. Campbell, Julia Y. Charles, Bryan Christensen, Athalia Christie, James R. Cope, Caitlin M. Cossaboom, Gretchen Cowman, Benjamin A. Dahl, Matthew M. Dahm, Mary Beth Danforth, Deena S. Disraelly, Christine Dubray, Jeffrey Eason, Jeanne Eckes, Denis FitzGerald, Laurie Forlano, Michael Garner, Eric Glassford, Cria O. Gregory, Jennifer Hannah, Yonette Hercules, Kevin Horahan, Brendan R. Jackson, Aaron D. Kofman, Inna Krapiunaya, David T. Kuhar, Joseph Lamana, James V. Lawler, Lily Lee, Kelcie Lever Landon, Julia Limage, John K. Lowe, Helen MacGregor, Brian M. Mazanec, Andrea M. McCollum, Emily L. McCutchen, Eva D. McLanahan, Jocelyn Mullins, Jennifer M. Nelson, Kate Noelte, Jennifer Renjel, Sheila Roy, Leanna Sayyad, Helga Scharf-Bell, Amy J. Schuh, Trevor R. Shoemaker, Kirstin Short, Emily Sims, Rachel L. Snyder, Christina F. Spiropoulou, Sydney R. Stein, Brittany Sunshine, Melissa Sutton, Julie M. Thompson, Alison M. Todres, Alexandra Tuttle, Axel A. Vazquez Deida, Aimee Williams, Emily C. Zielinski-Gutierrez, Joel M. Montgomery, David Fitter, 2026 U.S. Hantavirus Response Team, 2026 U.S. Hantavirus Response Team, Geoffrey C. Bartlett, Shelsy Cannon, Onieka Carpenter, Peter T. Cassell, Christina Cederberg, Jill M. D’Abrosca, Jeanette A. Dickinson, Dwight Ferguson, Artensie Flowers, Joseph M. Foss, Lydian Green, India Johns, John F. Koerner, Patrick J. Lindner, Erin Mullen, Christine M. Nappa, Anthony Paquette, Matthew Peitzman, Jenny Raspberry, Kimberley Ricketts, Catherine Roth, Cynthia Rubio, Erica Ruchard, Luis E. Torrens, Stephany Vento, Katie R. Watson, Margaret Wilk, Mallory Wright, Francisco Alvarado-Ramy, Beau B. Bruce, Heather Burke, Kevin Chatham-Stevens, Samuel J. Crowe, Chad H. Dowell, James C. Graziano, Anastasia Grindstaff, Inga Holmdahl, Reginald Jones, Rebecca Kahn, Cory M. Kokko, William T. Koval, Modinat Lawal, Sarah Leonard, Nekole S. Locke, Thomas C. McHale, Stephanie M. Morrison, Yoshinori Nakazawa, Rachel Nelson, Molly Nicholson, Scott W. Olesen, Meeyoung Park, Kara M. Pilote, Juliet R.C. Pulliam, Amanda Raziano, Erica Reott, Shahrokh Roohi, Laird Ruth, Tara Sealy, Melisa M. Shah, Alice Shumate, Dallas J. Smith, Rachel M. Smith, Jessica R. Spengler, Courtney Victor, Kendra Walker, Dennis Wang, Stephen R. Welch, Michael Wellman, Christina M. Winfield, Holly Howard, Vit Kraushaar, Eric M. Sergienko, Cameron Stainken, Alex Yu, Andrea Bessler, Skyler Brennan, Amanda Feldpausch, Julie Gabel, Robert Thornton, Farah S. Ahmed, Dereck D. Totten, Dennis Shoemaker, Justin Frederick, Lindsay Huse, Reed Thompson, Samir Koirala, Robin Williams, Angie M. Vasa, David Brett-Major, M. Jana Broadhurst, Angela Hewlett, Angela Ling, Elizabeth Schnaubelt, Michael C. Wadman, Katherine Willet, Wesley Zeger, Amanda Bartling, Stevie McGill, Amy Roden, William A. Fischer, Steven P. Dziura, Emily Lutterloh, Michelle Dethloff, Molly Howell, Brenton Nesemeier, Levi Schlosser, Michelle Ashby, Richard W. Fawcett, Carissa Heinige, Daphne Moffett, Dean Sidelinger, Janeana White, Briana O'Sullivan-Kovacs, Brendan Sullivan, Stephen White, Susan McClellan, Christopher A. Foster, Jeremy Langford, Sarah Mirabal, Joseph F. Uddo, Kevin Wilson, Evan Crook, Kacy Nowak, Bethelhem Kebede, Shawn Harper, James R. Moss, Caitlin S. Pedati, Denise C. Sockwell, Amber McCoy, BreeAnna Dell, Sargis Pogosjans, Michelle L. Holshue, Hanna Oltean

**Affiliations:** ^1^CDC Hantavirus Response; ^2^Epidemic Intelligence Service, CDC; ^3^New York State Department of Health; ^4^U.S. Department of State, Washington DC; ^5^Administration for Strategic Preparedness and Response, Washington DC; ^6^Maryland Department of Health, Baltimore, Maryland; ^7^Nebraska Department of Health and Human Services; ^8^California Department of Public Health, Richmond, California; ^9^Utah Department of Health and Human Services; ^10^Virginia Department of Health; ^11^University of Nebraska Medical Center, Omaha, Nebraska; ^12^Nebraska Public Health Laboratory, Omaha, Nebraska; ^13^Public Health – Seattle & King County, Seattle, Washington; ^14^Houston Health Department, Houston, Texas; ^15^Public Health Division, Oregon Health Authority, Portland, Oregon.; Administration for Strategic Preparedness and Response; Administration for Strategic Preparedness and Response; Administration for Strategic Preparedness and Response; Administration for Strategic Preparedness and Response; Administration for Strategic Preparedness and Response; Administration for Strategic Preparedness and Response; Administration for Strategic Preparedness and Response; Administration for Strategic Preparedness and Response; Administration for Strategic Preparedness and Response; Administration for Strategic Preparedness and Response; Administration for Strategic Preparedness and Response; Administration for Strategic Preparedness and Response; Administration for Strategic Preparedness and Response; Administration for Strategic Preparedness and Response; Administration for Strategic Preparedness and Response; Administration for Strategic Preparedness and Response; Administration for Strategic Preparedness and Response; Administration for Strategic Preparedness and Response; Administration for Strategic Preparedness and Response; Administration for Strategic Preparedness and Response; Administration for Strategic Preparedness and Response; Administration for Strategic Preparedness and Response; Administration for Strategic Preparedness and Response; Administration for Strategic Preparedness and Response; Administration for Strategic Preparedness and Response; Administration for Strategic Preparedness and Response; Administration for Strategic Preparedness and Response; Administration for Strategic Preparedness and Response; CDC; CDC; CDC; CDC; CDC; CDC; CDC; CDC; CDC; CDC; CDC; CDC; CDC; CDC; CDC; CDC; Epidemic Intelligence Service, CDC; CDC; CDC; CDC; CDC; CDC; CDC; CDC; CDC; CDC; CDC; CDC; CDC; CDC; CDC; CDC; CDC; CDC; CDC; Epidemic Intelligence Service, CDC; CDC; CDC; CDC; CDC; CDC; California Department of Public Health; California Department of Public Health; California Department of Public Health; California Department of Public Health; California Department of Public Health; Georgia Department of Public Health, Epidemic Intelligence Service, CDC; Georgia Department of Public Health; Georgia Department of Public Health; Georgia Department of Public Health; Georgia Department of Public Health; Kansas Department of Health and Environment; Kansas Department of Health and Environment; University of Kansas Medical Center; Douglas County Health Department; Douglas County Health Department; Douglas County Health Department; Nebraska Department of Health and Human Services; Nebraska Department of Health and Human Services; Nebraska Medicine; University of Nebraska Medical Center; University of Nebraska Medical Center; University of Nebraska Medical Center; University of Nebraska Medical Center; University of Nebraska Medical Center; University of Nebraska Medical Center; University of Nebraska Medical Center; University of Nebraska Medical Center; University of Nebraska Medical Center, Nebraska Public Health Laboratory; University of Nebraska Medical Center, Nebraska Public Health Laboratory; University of Nebraska Medical Center, Nebraska Public Health Laboratory; University of North Carolina; New York State Department of Health; New York State Department of Health; North Dakota Health and Human Services; North Dakota Health and Human Services; North Dakota Health and Human Services; North Dakota Health and Human Services; Deschutes County Health Services; Deschutes County Health Services; Deschutes County Health Services; Public Health Division, Oregon Health Authority; Public Health Division, Oregon Health Authority; Houston Health Department; Texas Department of State Health Services; Texas Department of State Health Services; Texas Department of State Health Services; University of Texas Medical Branch; U.S. Department of State; U.S. Department of State; U.S. Department of State; U.S. Department of State; U.S. Department of State; Utah Department of Health and Human Services; Utah Department of Health and Human Services; Arlington Health Department; Fairfax County Health Department; Virginia Department of Health; Virginia Department of Health; Virginia Department of Health; Grant County Health District; Public Health – Seattle & King County; Public Health – Seattle & King County; Career Epidemiology Field Officer Program, CDC, Washington State Department of Health; Washington State Department of Health.

**Summary**

**What is already known about this topic?**

Andes virus (ANDV), the only hantavirus known to be transmitted person-to-person, is endemic to areas of South America.

**What is added by this report?**

On May 4, 2026, CDC was notified of seven cases of severe acute respiratory illness, including three deaths, later confirmed to be caused by ANDV, on a cruise ship in the Atlantic Ocean. CDC collaborated with domestic and international partners to identify and monitor 88 U.S. residents with potential ANDV exposure. No secondary ANDV infections were identified among monitored contacts.

**What are the implications for public health practice?**

Rapid coordination supported identification, monitoring, and follow-up of exposed persons. Health care providers should consider ANDV infection among patients with compatible illness after travel to areas with endemic ANDV or exposure to a person with suspected or confirmed ANDV infection and promptly notify public health authorities of any cases.

## Abstract

Andes virus (ANDV), the only hantavirus known to be transmitted person-to-person, is endemic to areas of South America and can cause hantavirus pulmonary syndrome, which has a case-fatality rate of approximately 40%. On May 4, 2026, CDC was notified of seven cases of severe acute respiratory illness, including three deaths, among 147 persons aboard a cruise ship in the Atlantic Ocean; the illnesses were later confirmed to be caused by ANDV. No cases occurred among passengers who were U.S. residents. Ten of 28 U.S. resident passengers disembarked before ANDV infection was confirmed aboard the ship. On May 10, the remaining 18 U.S. resident passengers were repatriated to the National Quarantine Unit in Omaha, Nebraska, for risk assessment and monitoring, and where optimized advanced care was available, should symptoms develop. CDC and U.S. health departments monitored 60 additional U.S. residents potentially exposed on flights involving a passenger with symptomatic ANDV infection from the cruise, totaling 88 contacts across 27 jurisdictions. All 88 contacts completed 42 days of monitoring, with no secondary ANDV infections identified. Worldwide, local health authorities assessed and monitored approximately 653 potentially exposed persons from 33 countries. This response highlights the value of rapid coordination among partners to locate and monitor potentially exposed persons during an international outbreak to reduce the risk for ANDV transmission. Prompt notification to public health authorities can facilitate testing, patient care, contact identification, and implementation of measures to limit secondary transmission.

## Introduction

Andes virus (ANDV) is a New World hantavirus endemic to areas of South America (*1*). Human infection can occur through exposure to infected rodents or their excreta, and like illness caused by Sin Nombre virus (the most common cause of hantavirus pulmonary syndrome [HPS] in North America), illness caused by ANDV typically starts with nonspecific symptoms (*1*,*2*). ANDV is the only hantavirus known to spread person-to-person; transmission occurs via close prolonged contact or exposure to a symptomatic person’s body fluids (*1*). The incubation period is 4–42 days (*1*,*3*). Infection can cause mild illness or HPS, which can rapidly progress to respiratory failure and shock and might require advanced care, including extracorporeal membrane oxygenation (ECMO) (*4*). The reported HPS case-fatality rate is approximately 40% (*1*–*4*). No specific treatment or vaccine is available (*4*). The United States has reported one documented case of ANDV, an imported case in 2018 (*5*). This report describes the U.S. public health response and coordination among partners to identify and monitor potential contacts of persons with ANDV infection.

## Investigation and Findings

### Outbreak Detection 

On May 4, 2026, CDC received an International Health Regulations (IHR) notification of seven persons with severe acute respiratory illness aboard a cruise ship in the Atlantic Ocean, three of whom died. At the time of the report, the ship, which originally departed from Argentina, carried 147 persons, including 88 passengers and 59 crew members, representing 23 nationalities (*6*). The index patient developed symptoms on April 3 (*6*). Initial patients had fever and gastrointestinal symptoms, and four rapidly progressed to respiratory failure and shock (*6*,*7*). One critically ill passenger was medically evacuated to South Africa on April 27, where diagnostic testing confirmed ANDV infection on May 5 (*6*). No cases occurred among passengers who were U.S. residents. Onboard passengers were advised to remain in their cabins and maximize physical distancing (*7*). On May 10, the vessel docked in Tenerife, Canary Islands, Spain, where remaining passengers disembarked and received medical and public health assistance (Figure).

**FIGURE:**
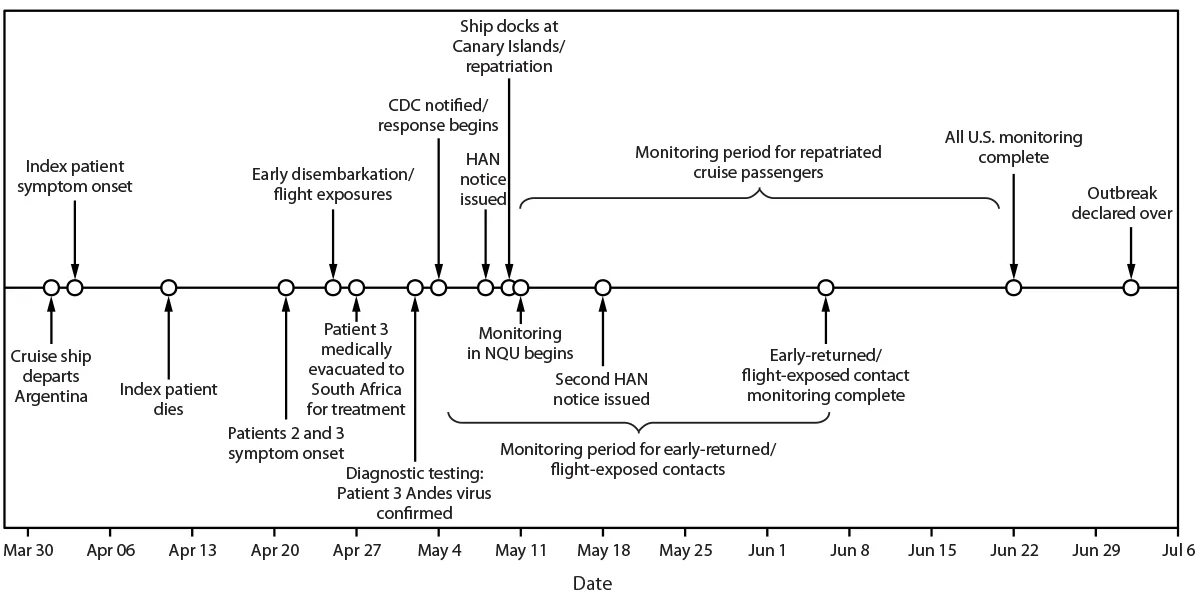
Timeline of hantavirus outbreak caused by Andes virus and public health measures — United States, 2026* **Abbreviations:** HAN = Health Alert Network; NQU = National Quarantine Unit. * Monitoring ended on June 5 (for one person), June 6 (for 69), and June 21 (for 18).

### U.S. Contact Identification

**Cruise contacts**. Twenty-eight U.S. residents traveled on the cruise (Supplementary Figure). Ten disembarked before ANDV transmission was identified; seven returned to their states of residence before May 2, and three remained abroad. Eighteen remained aboard the ship until its arrival in Tenerife. Through IHR notifications and information from federal partners, state and local health departments, and passengers, CDC located the seven potentially exposed U.S. residents who had returned to their states of residence and notified local public health jurisdictions for follow-up.

**Airline contacts.** Sixty U.S. resident contacts (as determined by authorities leading the investigations in South Africa or the Netherlands) were identified on two April 25 commercial flights boarded by a symptomatic cruise passenger later confirmed to have ANDV infection. The symptomatic passenger first boarded a flight from Saint Helena to Johannesburg, South Africa, carrying 11 U.S. residents (including eight cruise passengers). Eight U.S. residents (including six cruise passengers) from this flight returned to the United States for monitoring. The symptomatic passenger also boarded a flight from Johannesburg to Amsterdam, Netherlands, carrying 57 U.S. residents (no cruise passengers).* All 57 U.S. residents from this flight returned to the United States where they were monitored.

**Total contacts**. Overall, 88 unique U.S. resident contacts were identified, including 28 cruise passengers and 60 additional air travel contacts. Eighteen of these persons (cruise passengers) were transported to the United States (i.e., repatriated) for facility-based evaluation or monitoring, 66 (including seven cruise passengers) were monitored in home jurisdictions, and four (including three cruise and one air travel contact) were monitored abroad (Supplementary Figure).

### Laboratory Diagnostics

Before U.S. residents’ arrival at the National Quarantine Unit (NQU) in Nebraska, the Nebraska Public Health Laboratory (NPHL) rapidly validated an ANDV-specific real-time polymerase chain reaction (PCR) assay for testing of whole blood collected in ethylenediaminetetraacetic acid (EDTA) tubes. CDC recommended New World hantavirus serologic (immunoglobulin M [IgM] and immunoglobulin G [IgG]) testing of serum specimens from contacts with HPS-compatible symptoms, including fever, myalgia, chills, or acute gastrointestinal or respiratory symptoms (*8*,*9*). State and local health departments coordinated specimen collection from contacts with HPS-compatible symptoms; specimens were shipped to CDC for serologic testing for hantavirus. Repeat serologic testing was recommended >72 hours after symptom onset for persons whose initial specimen was collected ≤72 hours after symptom onset.

## Public Health Response

### Repatriation and Postarrival Evaluation

In coordination with a U.S. Department of State (DOS) biocontainment transport team, CDC conducted predeparture interviews with the 18 U.S. resident passengers in Tenerife to identify persons with ANDV-compatible symptoms and guide disembarkation, return flight seating, and repatriation procedures. The 18 passengers departed Tenerife on May 10 and arrived in the United States on May 11 aboard a DOS-chartered flight with biocontainment capability. Two physicians from the National Emerging Special Pathogens Training and Education Center (NETEC) provided technical guidance on infection prevention and control and clinical care during transport. Sixteen passengers were transported to the University of Nebraska Medical Center in Omaha, Nebraska; 15 were transported to NQU, and one asymptomatic passenger with an indeterminate ANDV PCR test result from a respiratory swab^†^ who was admitted to the U.S. Department of Health and Human Services (HHS) Region 7 Regional Emerging Special Pathogen Treatment Center (RESPTC) for evaluation. In addition, one symptomatic passenger and a travel companion were transferred to the HHS Region 4 RESPTC in Georgia for evaluation. Whole blood (EDTA) samples collected from the repatriated passengers were transferred to NPHL for PCR analysis; all test results were negative for ANDV. After receiving negative whole blood ANDV PCR and New World hantavirus serologic test results, all three passengers were transferred from RESPTCs to NQU on May 13 and May 15 (Supplementary Figure).

### Exposure Risk Stratification

CDC developed interim guidance (*9*) for managing ANDV contacts using a two-level risk-based approach (Table 1). Based on their presence aboard the vessel when patients with ANDV infection were symptomatic (April 3–May 10), all 28 U.S. cruise passengers were classified as having high-risk exposures.^§^ CDC and state and local health departments interviewed these 28 passengers to identify symptoms, confirm travel histories, characterize exposures to symptomatic patients, and support monitoring. Flight contact risk level was stratified by seat proximity to a person with symptomatic ANDV infection. Among 60 flight contacts, 12 (20%) and 48 (80%) had high- and low-risk exposures, respectively (Table 2).

**TABLE 1:** Interim public health monitoring measures for contacts of patients with Andes virus infection, by risk classification exposure criteria — U.S. jurisdictions, 2026

Risk classification	Exposure criteria	Public health monitoring measures
High risk	Being on board the affected cruise ship at any time from April 3 (the date of symptom onset for the index patient with ANDV infection) through the date of disembarkation of the exposed passenger cohort; answering “yes” to any of the exposure questions*; or being on an aircraft with a person with symptomatic ANDV infection and seated in the same row as the person or within two rows in front of or behind the person (high-risk zone)^†,§^	Twice-daily monitoring for fever and symptoms for 42 days after last potential exposure; modified activities to reduce close contact and potential exposures to others; movement and travel coordinated with public health authorities; and immediate isolation and evaluation if symptoms develop
Low risk	Being on an aircraft with a person with ANDV infection and seated outside of the high-risk zone	Daily symptom monitoring for 42 days after last potential exposure; no activity modifications or travel restrictions; jurisdictional coordination for travel; and immediate isolation and evaluation if symptoms develop

**Abbreviation:** ANDV = Andes virus.

* Exposure questions assessed direct contact with a symptomatic person or their body fluids, shared living or sleeping spaces, shared personal items, caregiving activities, and prolonged close contact (≤6 ft [1.8 m] for ≥15 cumulative minutes).

^†^ Aircraft crew members and anyone who provided direct assistance to the patient on board the aircraft were individually assessed for high-risk exposures.

^§^ Interim aircraft exposure criteria were updated during the response from a seat-based definition to a five-row zone comprising the patient’s row and two rows in front of and behind that row.

**TABLE 2:** Number and percentage of U.S. residents identified with potential high- or low-risk Andes virus exposure, by exposure setting — U.S. jurisdictions, 2026*

Exposure setting	Risk classification No. (%)		Total
	High risk^†^	Low risk^§^	
Cruise ship	28 (100)	0 (—)	**28**
Commercial flight from Saint Helena to Johannesburg, South Africa	0 (—)	3 (100)	**3^¶^**
Commercial flight from Johannesburg, South Africa, to Amsterdam, Netherlands	12 (21)	45 (79)	**57**
**Total**	**40 (45)**	**48 (55)**	**88**

* Four of the 88 U.S. residents did not return home during their monitoring period and were therefore not monitored by U.S. health departments.

^†^ All cruise passengers were classified as having high-risk exposures. Aircraft passengers seated in the same row as the infected traveler or within two rows in front of or behind the infected traveler were classified as having high-risk exposures.

^§^ Aircraft passengers seated more than two rows in front of or behind the infected traveler were classified as having low-risk exposures.

^¶^ Eight additional U.S. residents on the flight from Saint Helena to Johannesburg were also cruise ship passengers and are included in the cruise ship total but not the flight total.

### Monitoring and Testing

**Postarrival monitoring.** In collaboration with federal partners, CDC confirmed traveler details and identified 88 U.S. residents from 27 jurisdictions who required monitoring. CDC, the Administration for Strategic Preparedness and Response (ASPR), NQU, local jurisdictions, and international partners coordinated 42-day monitoring (upper end of the known ANDV incubation period) for all 88 U.S. resident passengers; 18 were monitored at NQU, four abroad through coordination with DOS and international authorities, and 66 in their home jurisdictions (Table 1) (*9*). The 18 contacts who disembarked in Tenerife were monitored at NQU for ≥21 days. ASPR deployed a team to manage NQU operations and logistics and to provide psychosocial support and other services, such as management of room service and personal item orders, to the passengers throughout the monitoring period.

After a ≥21-day monitoring period, ASPR coordinated with the U.S. Department of Homeland Security (DHS) and state and local health departments to transfer 12 contacts from NQU to their home jurisdictions. ASPR regional staff members worked with receiving jurisdictions to facilitate continued monitoring for the full 42 days and coordinated with NETEC and RESPTC on contingency plans to evaluate and manage persons who developed symptoms after returning home. The remaining six persons completed the 42-day monitoring period at NQU. Monitoring ended on June 5 (for one person), June 6 (for 69), and June 21 (for 18).

**Clinical consultations and testing.** CDC provided 33 clinical consultations to health departments and providers. In total, 127 PCR tests were completed for 21 contacts, including 18 repatriated cruise passengers, and three contacts who developed symptoms during monitoring; all ANDV PCR test results were negative. Serologic testing was completed for 27 contacts, including 18 cruise passengers, and nine contacts who developed symptoms during monitoring. Both IgM and IgG test results were negative for 26 persons; for one contact who might have been exposed to hantavirus previously, only the IgG test result was positive.

### U.S. and International Coordination

Through the HHS Disaster Leadership Group, ASPR led federal interagency response coordination among DHS, DOS, CDC, and other HHS partners. Two CDC Health Alert Network (HAN) notices informed clinicians and health departments about case identification, testing, and biosafety (2026 Multi-Country Hantavirus Cluster Linked to Cruise Ship | HAN | CDC; 2026 Hantavirus Outbreak: Testing for Potential Infection | HAN | CDC). Frequent partner and press briefings and state, tribal, local, and territorial health department calls disseminated information. CDC and ASPR coordinated outbreak response activities with U.S. agencies, international partners (World Health Organization, Pan American Health Organization, and European Centre for Disease Prevention and Control), and international government public health agencies. CDC also developed a risk assessment to help guide domestic monitoring and response planning. The One CDC Data Platform, which supports data collection, integration, analysis, and visualization, facilitated secure sharing of jurisdictional monitoring data and tracking of daily updates. This activity was reviewed by CDC, deemed not research, and conducted consistent with applicable federal law and CDC policy.^¶^

## Discussion

By June 2, 2026, six additional ANDV outbreak cases had been identified (for a total of 13 cases, including three deaths) worldwide (*7*). All cases occurred among persons aboard the cruise ship (*7*). Globally, health authorities assessed and monitored approximately 653 persons from 33 countries (*7*). Because approximately 4 weeks had elapsed between the index patient’s symptom onset and confirmation of ANDV infection, potentially exposed U.S. travelers had dispersed across multiple U.S. and international jurisdictions. Non-U.S. resident contacts were monitored by health officials in their respective countries. All 88 U.S.-associated contacts completed monitoring, and no secondary ANDV infections associated with this cohort were identified. One contact was presumed to have had a previous New World hantavirus infection unrelated to the outbreak, highlighting the importance of interpreting serologic results in the context of epidemiologic data.

The absence of secondary infections among monitored U.S. contacts should not be interpreted as evidence that no transmission risk existed. Close quarters and shared spaces aboard the ship created numerous opportunities for exposure. Additional studies might help identify factors associated with person-to-person transmission of ANDV.

Health care providers should obtain a thorough travel history and consider ANDV in any acutely ill patient with compatible illness who has been in an area where ANDV infection is endemic or in close contact with someone with suspected or confirmed ANDV infection, irrespective of contact location. ANDV can be difficult to distinguish clinically from Sin Nombre virus infection because early signs and symptoms are nonspecific. However, because ANDV (unlike Sin Nombre virus) can be transmitted person-to-person, prompt notification to public health authorities of persons with potential ANDV infection can facilitate testing, contact identification, and implementation of measures to reduce secondary transmission. In this outbreak, enhanced contact monitoring facilitated rapid clinical evaluation with ability to transfer patients to ECMO-equipped centers, if necessary. Because viremia can precede symptom onset, PCR testing can facilitate early identification of infections (*10*). Modified activity recommendations and isolation were intended to reduce transmission risk during early illness, when person-to-person transmission had been reported (*1*,*2*). Multijurisdictional investigations can be complex, particularly for outbreaks involving cross-jurisdictional or international travel. In this outbreak response, rapid coordination among domestic and international public health and other partners reduced the risk for additional infections.

## Footnotes

* Investigation revealed that a symptomatic non-U.S. cruise passenger who was later confirmed to have ANDV infection briefly boarded then disembarked from the flight from Johannesburg to Amsterdam.

† The asymptomatic passenger’s PCR result came from testing performed at a reference laboratory in the Netherlands on a specimen collected before disembarkation.

§ CDC determined that presence on board the cruise ship constituted a high-risk exposure because of the likelihood of prolonged close contact among passengers and crew, shared living spaces, and the potential for recall bias when assessing exposures and symptom onset.

¶ 45 C.F.R. part 46.102(l)(2), 21 C.F.R. part 56; 42 U.S.C. Sect. 241(d); 5 U.S.C. Sect. 552a; 44 U.S.C. Sect. 3501 et seq.
